# Safety Assessment of Extracellular Vesicle-Based Therapy in Regenerative Dentistry

**DOI:** 10.3390/ijms27020798

**Published:** 2026-01-13

**Authors:** Bing-Huan Chuah, Jia-Xian Law, Xin-Fang Leong, Kok-Lun Pang, Yan-Rou Farm, Masfueh Razali, Sook-Luan Ng

**Affiliations:** 1 Department of Craniofacial Diagnostics and Biosciences, Faculty of Dentistry, Universiti Kebangsaan Malaysia, Kuala Lumpur 50300, Malaysia; p140783@siswa.ukm.edu.my (B.-H.C.); leongxinfang@ukm.edu.my (X.-F.L.); p145608@siswa.ukm.edu.my (Y.-R.F.); 2 Department of Tissue Engineering and Regenerative Medicine, Faculty of Medicine, Universiti Kebangsaan Malaysia, Kuala Lumpur 56000, Malaysia; lawjx@hctm.ukm.edu.my; 3 Jeffrey Cheah School of Medicine & Health Sciences, Monash University Malaysia, Petaling Jaya 47500, Malaysia; koklun.pang@monash.edu; 4 Department of Restorative Dentistry, Faculty of Dentistry, Universiti Kebangsaan Malaysia, Kuala Lumpur 50300, Malaysia; masfuah@ukm.edu.my

**Keywords:** extracellular vesicles, dental pulp stem cells, mesenchymal stem cells, regenerative dentistry, angiogenesis, bone regeneration, periodontitis, immunomodulation, scaffold integration

## Abstract

Extracellular vesicle (EV)-based therapies have emerged as promising, cell-free approaches for dental tissue regeneration. This narrative review integrates mechanistic insights, therapeutic efficacy data, and safety and delivery considerations from in vitro and in vivo studies to elucidate the molecular mechanisms by which EVs, particularly those from dental pulp stem cells (DPSCs) and mesenchymal stem cells (MSCs), drive regenerative processes via key signalling axes (PI3K/Akt, MAPK, BMP/Smad, and Hedgehog). Preclinical studies demonstrate that unmodified and engineered EVs enhance odontogenic differentiation, angiogenesis, bone repair, and immunomodulation in models of pulp regeneration, alveolar bone defects, osteonecrosis, and periodontitis. Isolation and purification methodologies were also evaluated, comparing ultracentrifugation, size-exclusion chromatography, and density-cushion approaches, and discussing how protocol variations affect EV purity, dosing metrics, and functional reproducibility. Early-phase clinical evaluations report only low-grade transient adverse events, underscoring a generally favourable safety profile. Despite these encouraging results, significant challenges remain: heterogeneity in EV cargo composition, lack of standardised potency assays, and incomplete long-term safety data. The review highlights the urgent need for rigorous, harmonised regulatory frameworks and robust quality control measures to ensure that EV-based modalities can be translated into safe, effective, and reproducible therapies in regenerative dentistry.

## 1. Introduction

**Figure 1:**
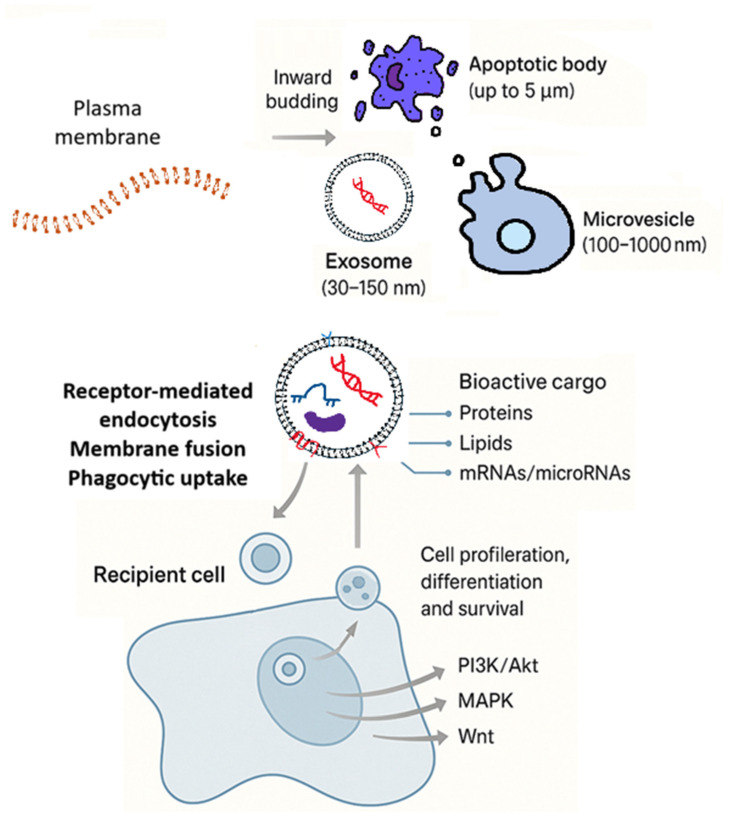
Overview of EV types, formation, and uptake. Biogenesis of exosomes [via inward budding], microvesicles [via outward budding], and apoptotic bodies [from dying cells]. The image illustrates EV uptake by recipient cells and downstream signalling pathways [PI3K/Akt, MAPK, Wnt] involved in regulating proliferation, differentiation, and survival. Bioactive cargo is represented by coloured icons for proteins, lipids, and nucleic acids.

Extracellular vesicles (EVs) are heterogeneous, membrane-enclosed particles released by nearly all cell types and are primarily categorised as exosomes (30–150 nm, derived from the endosomal pathway), microvesicles (100–1000 nm, generated by direct plasma membrane budding), and apoptotic bodies (up to 5 µm, formed during programmed cell death) [1]. Their biogenesis is governed by distinct molecular machinery; exosomes arise via the inward budding of multivesicular bodies and subsequent fusion with the plasma membrane, while microvesicles form through outward membrane blebbing [2]. Apoptotic bodies, on the other hand, are produced during the late stages of apoptosis through cell shrinkage, chromatin condensation, and membrane blebbing, followed by fragmentation of the dying cell into membrane-bound vesicles containing cellular organelles and nuclear material [3] (Figure 1).

The various types of EVs, along with their formation and uptake mechanisms, are depicted in Figure 1. EVs encapsulate a complex cargo of proteins, lipids, and nucleic acids, including messenger RNAs (mRNAs), microRNAs (miRNAs), and long non-coding RNAs (lncRNAs), which modulate recipient cell functions by altering intracellular signalling pathways such as Phosphoinositide 3-Kinase (PI3K/Akt), Mitogen-Activated Protein Kinase (MAPK), and Wnt (Wingless/Integrated Signalling Pathway) [4]. The targeted delivery of this bioactive cargo occurs through receptor-mediated endocytosis, membrane fusion, and phagocytic uptake, enabling EVs to orchestrate specific cellular responses [4]. Research indicates that EVs derived from dental pulp cells, for example, carry specific miRNAs and proteins that activate signalling cascades such as PI3K/Akt and MAPK [5], which are essential for odontogenic differentiation and angiogenesis [6]. Moreover, EVs isolated from MSCs have been shown to regulate the inflammatory microenvironment by inducing M2 macrophage polarisation, thereby reducing inflammatory responses while simultaneously promoting osteogenesis and vascular remodelling critical for periodontal regeneration [7]. Notably, exosomes from stem cells of the apical papilla have demonstrated the ability to stimulate craniofacial bone repair [4] through the activation of Wnt and BMP (bone morphogenetic protein)/Smad (suppressor of mother against decapentaplegic family proteins) pathways, underscoring the multifaceted role of EVs in orchestrating molecular events that drive tissue regeneration [5,8,9]. These bioactive vesicles bypass many of the limitations associated with traditional cell-based therapies, such as donor variability, senescence, and immune rejection, thus presenting a more reliable and targeted approach to tissue regeneration [10,11].

This review aims to critically analyse original research to elucidate the molecular mechanisms underlying EV-based therapies in regenerative dentistry, with a particular emphasis on safety and standardisation. By analysing how EV cargo modulates key signalling pathways such as PI3K/Akt, MAPK, BMP/Smad, and Wnt [12], the review seeks to clarify the direct links between these molecular events and clinical regenerative outcomes. It will also evaluate current methodologies for EV isolation, purification, and characterisation, highlighting how variations in these processes impact both efficacy and safety, specifically addressing concerns such as immunogenicity and unintended cellular responses. Furthermore, the review will critically assess preclinical and clinical evidence on EV-mediated tissue repair, wound healing, and bone regeneration to provide a comprehensive framework for the safe integration of EV-based therapies into clinical practice. Ultimately, this synthesis of research findings aims to inform future translational strategies and standardisation protocols, facilitating the development of reliable, cell-free therapeutic modalities to harness precise molecular pathways for enhanced regenerative outcomes in dentistry.

## 2. Mechanisms of Action of EVs in Regenerative Dentistry

### 2.1. EVs as Mediators of Cellular Communication

In periodontal regeneration, EVs derived from periodontal ligament stem cell-derived exosomes (PDLSC-Exo) have been shown to modulate osteogenic differentiation via the miR-34c-5p/special AT-rich sequence-binding protein 2 (SATB2)/extracellular signal-regulated kinase (ERK) axis [13] while concurrently delivering miR-17-5p to activate vascular endothelial growth factor receptor (VEGF/VEGFR)-mediated angiogenesis [14] and PI3K/Akt-dependent suppression of nuclear factor kappa-light-chain-enhancer of activated B cells (NF-κB)-driven inflammation [15]. Mesenchymal stem cell-derived extracellular vesicles (MSC-EVs) likewise attenuate macrophage-mediated inflammation by blocking NF-κB activation through inhibition of inhibitor of nuclear factor kappa B alpha (IκBα) phosphorylation [16], reducing tumour necrosis factor alpha (TNF-α) [16] and interleukin (IL-6) secretion [17,18], a beneficial immunomodulatory effect that nonetheless poses a risk of impaired host defence if dosing is not carefully controlled [19,20,21].

However, these regenerative pathways carry inherent safety considerations. Hyperactivation of Wnt/beta-catenin (β-catenin) signalling has been implicated in tumourigenesis [22,23,24] and ectopic calcification [25,26], underscoring the necessity for precise dose titration and rigorous cargo characterisation to avoid oncogenic risks. Similarly, excessive modulation of NF-κB or ERK pathways can lead to off-target immunosuppression [27] or fibrosis [15,28,29]. Moreover, cargo heterogeneity, variability in miRNA, protein, and lipid composition among EV batches, can result in inconsistent pathway engagement and unpredictable biological responses [30,31].

### 2.2. Source Cells and Molecular Signalling Mediated by EVs

EVs for dental regeneration have been sourced from multiple stem and progenitor cell populations, each offering distinct cargo profiles and regenerative capacities. DPSC-derived EVs, harvested by differential ultracentrifugation of conditioned medium from extracted third molars [32], are enriched in odontogenic proteins (e.g., DSPP, DMP1) and miRNAs such as miR-196a. In a rat mandibular defect model, these EVs upregulated RUNX2 and alkaline phosphatase in the jawbone and accelerated bone repair [33].

Periodontal ligament stem cell-derived exosomes (PDLSC-Exo), likewise isolated by ultracentrifugation, deliver miR-34c-5p to inhibit SATB2/ERK signalling [34], modulating osteogenic differentiation, and promoting VEGF/VEGFR-mediated angiogenesis through exosome-mediated transfer of VEGFA regulated by miR-17-5p [14] while concurrently attenuating inflammation via NF-κB suppression and reduced TNF-α/IL-6 secretion [35,36,37]. Stem cells from human exfoliated deciduous teeth (SHED) produce EVs that, after purification by size-exclusion chromatography, enhance HUVEC proliferation, migration, and tube formation [38]. These effects are maintained under hyperglycaemic conditions through GATA2 and CD31 upregulation [34]. In mouse periodontitis models, SHED-EVs restore alveolar bone comparably to SHED transplantation without inducing target-cell apoptosis [34].

Gingival MSC (GMSC)-derived EVs cross-regulate NF-κB and Wnt/β-catenin pathways in PDLSCs, inhibiting porphyromonas gingivalis-derived lipopolysaccharide (LPS)-induced NF-κB activation and upregulating Wnt/β-catenin to promote osteogenesis under both homeostatic and inflammatory conditions [39].

Among non-dental sources, umbilical cord MSCs (UC-MSCs) yield approximately fourfold more EVs per cell than bone marrow MSCs under identical culture conditions, highlighting their scalability for clinical-grade EV production [40]. Finally, exosomes from hiPSC-derived endothelial cells, enriched in miR-199b-5p, enhance HUVEC proliferation, migration, and tube formation via Jagged1/VEGFR2 signalling and promote neovascularisation in hind-limb ischaemia models, illustrating the versatility of iPSC-EVs for vascularised tissue repair [41].

#### Donor Age, Cell State, and Microenvironment

EV cargo reflects the physiological state of the parent cell; hence, donor age, replicative history, and inflammatory or metabolic microenvironments can significantly alter EV composition and downstream regenerative potency. Ageing is associated with shifts in EV abundance and cargo, such as miRNA profiles in circulation and in EVs released by somatic tissues, consistent with the concept that EVs transmit state signals to recipient cells. In a bone-relevant context, muscle-derived miRNA changes have been linked to age-associated depletion of bone stem or progenitor pools [42], supporting a mechanism by which systemic ageing alters osteogenic capacity via EV-mediated signalling. Conversely, EVs from neonatal or younger donors can exhibit pro-rejuvenation activity. Neonatal umbilical cord MSC-derived EVs were shown to restore functional deficits in aged bone marrow MSCs through transfer of proliferative and repair signals, including proliferating cell nuclear antigen (PCNA)-associated mechanisms [43], and to attenuate age-related degeneration in vivo, showing that donor age can qualitatively change EV therapeutic effects. Beyond chronological age, the parent-cell microenvironment is an important aspect of altering EV function, as inflammatory or lineage-specific conditioning can enrich EV cargo linked to immunomodulation or osteogenesis and increase the need for batch-level cargo and potency characterisation, because conditioning can increase the variability between preparations [44].

### 2.3. EV-Mediated Tissue Regeneration in Dentistry

**Figure 2:**
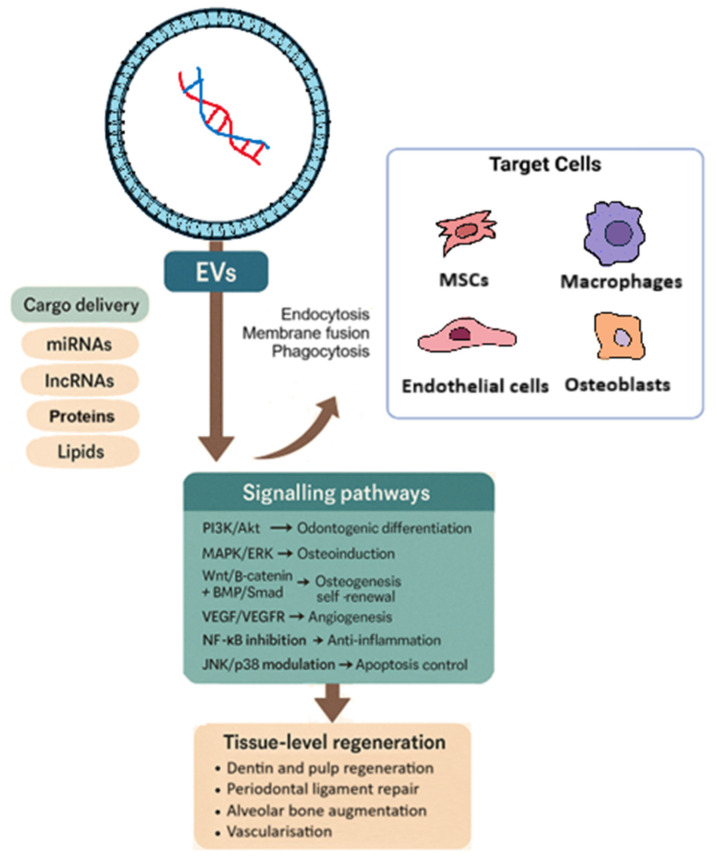
EV-mediated regenerative mechanisms in dentistry. EVs shuttle proteins, lipids and RNAs into target cells (MSCs, macrophages, endothelial cells, and osteoblasts) via endocytosis, membrane fusion, or phagocytosis, activating PI3K/Akt, MAPK/ERK, Wnt/BMP/Smad, VEGF/VEGFR, NF-κB, and JNK/p38 pathways to promote odontogenesis, osteogenesis, angiogenesis, immunomodulation, and ultimately dentin/pulp, periodontal, and alveolar bone regeneration.

The pathways or mechanisms, based on both preclinical and clinical models, in dental regeneration are depicted in Figure 2. EVs have emerged as potent mediators of tissue regeneration in dental contexts, coordinating cell proliferation, differentiation, migration, and immunomodulation via several conserved signalling cascades. In particular, EVs activate the Wnt/β-catenin [45], Cdc42 [46], ERK/MAPK [47], PI3K/Akt [48], and NF-κB [49] pathways to orchestrate complex repair processes in dental pulp cells, periodontal ligament cells, osteoblasts/pre-osteoblasts, and endothelial cells [45,46,47,48,49].

In osteogenic and dentinogenic models, human embryonic stem cell (ESC)-derived EVs have been shown to transport miR-196a to pre-osteoblasts [50], thereby enhancing differentiation and upregulating key osteogenic markers, including Runx2, alkaline phosphatase, and osteocalcin, in alveolar bone explants [51,52,53]. These findings underscore the ability of ESC-EVs to mimic developmental cues and drive bone-forming programmes ex vivo.

Expanding upon mechanistic pathways, exosomes harvested from stem cells of human exfoliated deciduous teeth (SHED-Exo) co-activate Wnt/β-catenin and BMP signalling in periodontal ligament stem cells, resulting in robust osteogenic commitment in vitro [54]. Exosomes derived from the apical papilla (SCAP-Exo) deliver active Cdc42 protein directly into endothelial cells, triggering cytoskeletal reorganisation, filopodia formation, and angiogenic sprouting, critical steps for generating vascularised craniofacial tissues [46]. Mesenchymal stem cell-derived EVs (MSC-EVs) further contribute to regenerative outcomes by exerting anti-inflammatory effects alongside the promotion of new bone formation in periodontal defect models [55]. Additionally, activation of the canonical Wnt/β-catenin pathway enhances stem cell self-renewal and osteogenic gene transcription [54], while Cdc42 signalling controls cytoskeletal dynamics and endothelial motility [46]. The ERK/MAPK and PI3K/Akt pathways coordinate cell survival and proliferation during repair, and modulation of NF-κB activity shapes the immunomodulatory and matrix-remodelling environment necessary for tissue regeneration [49]. By engaging these pathways in concert, EVs recreate developmental signalling networks to accelerate dental tissue repair.

Translational studies also highlight the utility of these insights in engineered constructs. Zhao et al. (2022) embedded periodontal ligament-derived EVs within a gelatin–alginate hydrogel scaffold and reported a 15 percent increase in alveolar bone volume at four weeks, along with more extensive Masson’s trichrome-stained bone formation compared with hydrogel alone [56]. Lu et al. (2024) extended this approach by incorporating SHED-Exo into a gelatin methacryloyl (GelMA) matrix to enhance odontogenic differentiation of dental pulp stem cells, achieving significantly greater dentin matrix deposition and upregulation of dentin sialophosphoprotein (DSPP) [6].

Finally, combinatorial strategies that integrate pro-angiogenic growth factors, such as VEGF, bFGF, and PDGF, into EV-laden scaffolds have demonstrated synergistic enhancements in neovascularisation and osteogenesis in dental defect models [54,56].

## 3. Safety Concerns in EV-Based Therapies

### 3.1. Immunogenicity of EVs

EV-based therapies raise important safety concerns, primarily related to immune activation, biodistribution, and off-target effects. Key immunogenicity outcomes include complement activation, cytokine release, and the generation of anti-EV antibodies upon repeated administration [57,58]. These risks are amplified in vulnerable populations and underscore the need to assess both the magnitude and duration of host immune responses prior to clinical application.

Patient-specific factors, including age, health status, and comorbidities, play a critical role in modulating these safety outcomes. Age-related physiological changes, such as cellular senescence and diminished regenerative potential, can alter EV biodistribution and clearance, increasing the likelihood of suboptimal efficacy or heightened inflammatory responses [58,59,60,61]. Similarly, patients with chronic diseases or immunocompromised conditions may exhibit altered pharmacokinetics or reduced tolerability to allogeneic EVs, raising the risk of exaggerated immune responses [10,57,58,62]. A thorough evaluation of each patient’s immune profile and clinical background is thus essential for tailoring dosing regimens and minimising adverse events [63].

While EVs, particularly those derived from MSCs, are generally regarded as having low immunogenicity [11,64], this profile is not uniform. It varies significantly based on cellular origin, donor compatibility, and purification method [50,60,61,64]. Autologous EVs tend to carry lower immunological risk, whereas allogeneic or xenogeneic EVs may present surface antigens that trigger host immune reactions [57,58]. In addition, the method of EV isolation can co-purify immunostimulatory contaminants such as nucleic acids and lipoproteins, which may contribute to complement activation or Toll-like receptor signalling [11,58,65,66]. These findings support the need for standardised isolation techniques and stringent quality control measures to reduce batch-to-batch variability and ensure clinical safety.

EV surface components and cargo, including membrane proteins, tetraspanins, RNA species, and phosphatidylserine, can interact with pattern recognition receptors (PRRs) on immune cells. These molecular features may facilitate macrophage uptake, promote cytokine secretion, or activate complement cascades, potentially leading to systemic inflammation [58,63]. Rational design strategies such as EV surface engineering, preconditioning of parent cells, or cargo modulation may help reduce these immunogenic epitopes and improve tolerability [67,68].

The route of administration, delivery matrix, and dosing schedule are equally critical in shaping immune responses. For dental applications where local injection to the intragingival, periodontal pocket, or intrabony delivery is feasible, immunotoxicity considerations must account for the specialised immune landscape of the gingival barrier, which is continuously exposed to tooth-associated biofilm and mechanical microtrauma. EVs actively modulate the oral immune microenvironment by regulating macrophage polarisation, Th17/Treg balance, and NF-κB-driven cytokine signalling in gingivitis and periodontitis, underscoring the need to evaluate immunogenicity within oral-specific inflammatory contexts [69]. In a large human cohort, the gingival interface in health was characterised by a predominance of T cells, relatively few B cells, and a substantial presence of granulocytes and neutrophils, and antigen-presenting cell networks. In periodontitis, this immune architecture shifts further with increased neutrophils and heightened IL-17 responses within the CD4+ T-cell compartment [70]. These features are relevant because EV surface molecules and co-isolated contaminants, including nucleic acids, LPS, and lipoproteins, may engage pattern recognition pathways on resident epithelial cells, neutrophils, macrophages, and dendritic cells, potentially amplifying local cytokine release or altering clearance kinetics after injection. In keeping with this context dependence, dental-tissue MSC exosomes have been evaluated directly in inflammatory periodontal settings, as gingival MSC-derived exosomes suppress inflammatory signalling and restore the regenerative potential of PDLSCs in an inflammatory microenvironment [71], and TNF-α primed gingiva-derived MSC exosomes promote anti-inflammatory macrophage polarisation and reduce inflammatory bone loss in an LIP model [7]. Evidence therefore suggests that local delivery in the oral cavity may not be immunologically neutral; therefore, dental EV products should be evaluated in preclinical systems that incorporate oral-relevant immune cues such as biofilm products, IL-17, and neutrophil-rich inflammation, and local APC networks, to better predict tolerability and the therapeutic window.

Repeated systemic injections of unmodified EVs may lead to immune memory and accelerated clearance, while localised delivery systems, such as hydrogels, can enhance tissue retention and reduce systemic exposure [6,72]. As shown by Lu et al. (2024), preconditioning SHEDs in odontogenic medium generated OM-EVs enriched in pro-regenerative factors that activated the AMPK/mTOR pathway [6]. When embedded in GelMA hydrogel, these OM-EVs improved safety by ensuring sustained, site-specific delivery and significantly enhanced dentinogenesis in vitro and in vivo [6].

In periodontitis and other oral conditions, EV-based therapy must also account for craniofacial tissue-specific factors, local immune microenvironments, and angiogenic variability among patients [56,73]. For example, Cai et al. (2023) emphasised the need for immune-modulating EV formulations tailored to individual profiles in periodontal therapy [74], while Fuloria et al. (2021) highlighted the importance of customising MSC-EV therapy for regenerative applications across diverse clinical populations [75].

**Figure 3:**
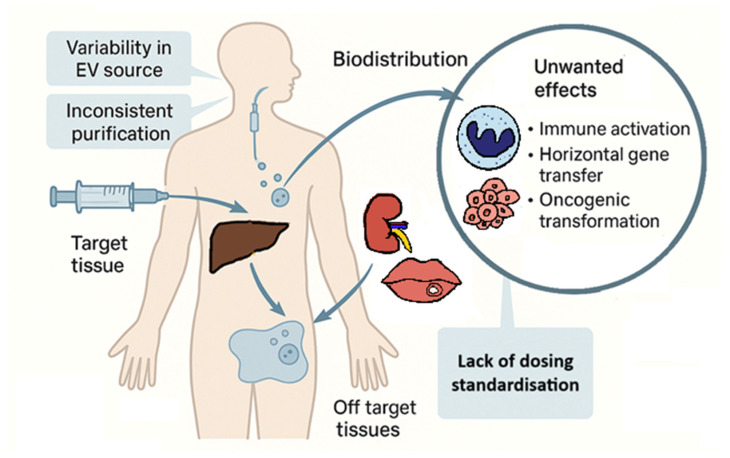
Safety concerns in extracellular vesicle (EV)-based therapies, particularly within the context of regenerative dentistry. Key risks include off-target effects, potential immunogenicity, transmission of undesirable cargo, and tumourigenic potential.

To advance the safe clinical translation of EV-based therapies, an integrated approach is required. This includes matching EV sources to patient immune status, optimising purification methods, controlling delivery kinetics, and understanding immune engagement pathways [55,65,66,72]. An overview of safety considerations in EV-based therapies is presented in Figure 3.

### 3.2. Risk of Tumourigenesis

EV-based therapies carry the risk of inadvertently promoting cancer progression rather than initiating de novo tumourigenesis. Through horizontal transfer of oncogenic proteins, RNAs, and other bioactive molecules, EVs can activate key signalling cascades in recipient cells, enhancing proliferation, migration, invasion, stemness, and metastatic potential. Although true tumourigenesis implies the initial transformation of normal cells, the bulk of current evidence implicates EVs in accelerating the growth and spread of pre-existing malignant cells. Consequently, evaluating the pro-tumourigenic capacity of therapeutic EVs, particularly in patients with occult or existing cancers, is essential to safe clinical translation.

Multiple studies have dissected the pathways and EV sources responsible for these effects. Gu et al. (2016) showed that human bone marrow MSC-derived exosomes stimulate AKT signalling in human HGC-27 gastric cancer cells, driving epithelial–mesenchymal transition, increased expression of stemness markers (OCT4, SOX2, and Lin28B), and enhanced ex vivo tumourigenicity [76]. Qi et al. (2017) found that MSC exosomes activate Hedgehog signalling in human MG63 osteosarcoma and human SGC7901 gastric cancer cells, a proliferative effect reversible by Hedgehog pathway inhibitors, underscoring the role of developmental pathways in EV-mediated cancer progression [77].

More recently, Qi et al. (2023) reported that MSC microvesicles conditioned by Helicobacter pylori carry elevated thrombospondin-2, which, upon transfer to MGC-803 gastric cancer cells, promotes in vitro proliferation, invasion, and in vivo tumour growth with peritoneal metastasis in mouse xenografts [78]. Similar mechanisms operate in oral tissues: exosomes from MSCs derived from dysplastic oral leukoplakia and oral carcinoma in in vitro models are enriched in miR-8485, which accelerates proliferation, migration, and invasion and downregulates p53 expression—effects reversed by blocking exosome secretion, suggesting that EVs from premalignant lesions may fuel malignant transformation and recurrence in the oral cavity [79].

### 3.3. Toxicity of EV Cargo

EV cargo can pose significant acute toxicity risks, particularly haematotoxicity and cytotoxicity, that must be addressed before clinical application. Procoagulant lipids and proteins on EV surfaces catalyse clotting cascades: for instance, MSC-EVs bearing phosphatidylserine accelerate tenase and prothrombinase assembly, shortening plasma clotting times by 25–30% and carrying factor V, prothrombin, von Willebrand factor, CD9, and kallikrein, all of which amplify thrombin generation and could precipitate thrombosis in susceptible patients [80].

Similarly, EVs from umbilical cord MSCs generate activated factor X (0.023 ± 0.017 nM FXa per 106 EVs), an effect tied directly to tissue factor content since a blocking antibody reduced FXa production by over 85% [81]. Moreover, plasma EVs isolated from multiple myeloma patients exhibit a nearly two-fold increase in endogenous thrombin potential and a 60% rise in peak thrombin generation, effects attributable to large EVs enriched in tissue factor and procoagulant phospholipids, underscoring that both EV source and subpopulation critically determine haematologic safety profiles [82]. Beyond coagulation, damage-associated molecular patterns (DAMPs) such as HMGB1 carried by EVs from LPS-activated macrophages can trigger RAGE-mediated NLRP3 inflammasome activation in hepatocytes, inducing caspase-1-dependent pyroptosis and contributing to organ injury in sepsis models [83]. Although some of these pathways lie outside dental applications, they illustrate that EV preparations must be rigorously screened for pro-inflammatory and pro-death signals to avoid acute toxicity.

In addition to these immediate effects, EV-mediated transfer of pathogenic microRNAs can drive tissue-specific injury and chronic dysfunction. For example, EVs from steatotic hepatocytes enriched in miR-1 suppress KLF4 and activate NF-κB in endothelial cells, upregulating ICAM-1 and VCAM-1, promoting leukocyte adhesion, and accelerating atherogenesis in ApoE^−^/^−^ mice [84,85]. Podocyte-derived EVs carrying miR-149 and miR-424 induce apoptosis in proximal tubular cells via BCL2 downregulation and caspase-3 activation, contributing to acute tubular injury in vitro [7]. Additionally, systemic elevation of miR-424 in diabetic nephropathy rat models attenuated renal lesions by targeting Rictor and modulating AKT signalling, highlighting that the same cargo can have context-dependent effects [7].

In the context of regenerative dentistry, procoagulant activity could impair pulp revascularisation, DAMP-driven inflammation might disrupt periodontal healing, and unwanted miRNA transfer could compromise local cell viability. Critically assessing cargo heterogeneity, defining safe concentration thresholds, and developing strategies to deplete or neutralise toxic components will be essential for translating EV therapies into safe, effective dental regenerative treatments.

### 3.4. Contamination Risks

EV preparations are prone to co-isolating non-EV particles, most notably lipoproteins, abundant serum proteins, and microbial contaminants, which compromise both functional purity and safety [86]. Albumin and ApoB-positive lipoproteins frequently persist in isolates, increasing the risk of unpredictable bioactivity and inflammatory reactions in clinical use [87]. Viral and bacterial components further exacerbate this problem: enveloped viruses such as HIV-1 can co-purify with exosomes in standard density gradients, posing a risk of inadvertent pathogen transfer [88], while bacterial endotoxins, up to 1.2 × 10^13^ LPS units per 10^10^ EVs in *P. gingivalis* vesicle preparations, can trigger potent TLR4-mediated inflammation if not removed [89,90]. Mycoplasma [91], a common cell-culture contaminant, releases EV-like particles that infiltrate human cells and modulate host proteomes, underscoring the necessity of routine mycoplasma surveillance and elimination during EV production [92,93].

Common isolation techniques each carry intrinsic limitations that favour specific contaminant classes. Precipitation methods using PEG tend to co-pellet serum protein aggregates and high-density lipoproteins, while size-based methods alone, such as size-exclusion chromatography, fail to fully clear these contaminants without additional density-based steps [94,95]. Differential ultracentrifugation reduces bulk impurities but cannot eliminate smaller lipoprotein particles or tightly bound protein complexes [95]. These technical shortcomings highlight the need for orthogonal purification strategies, combining density cushions, affinity capture, and sequential filtration, to achieve reproducible EV purity [96,97]. Moreover, comprehensive contaminant profiling, including markers such as ApoB, viral capsid proteins, and endotoxin levels, is essential for quality control and to ensure that therapeutic EV products meet stringent safety standards [98,99,100]. In the context of regenerative dentistry, residual lipoproteins or microbial components could provoke pulp inflammation, interfere with scaffold integration, or undermine immunomodulatory benefits, so rigorous multimodal purification and validation remain critical prerequisites for clinical translation.

## 4. Discussion and Future Challenges

### 4.1. Advancements in EV-Based Therapies

Recent years have witnessed significant progress in engineering EV-based therapies to enhance dental tissue regeneration. Early-phase clinical investigations have begun to explore the use of EVs in craniofacial bone regeneration.

One promising strategy involves embedding EVs within biocompatible hydrogels to improve their retention and sustained release at defect sites. For example, Zhang et al. (2020) demonstrated that fibrin gels loaded with dental pulp stem cell-derived EVs promoted rapid neovascularisation, collagen I/III deposition, and angiogenic factor release under nutrient-deficient conditions in vitro, suggesting that such constructs can overcome the rapid clearance of EVs and support vascularised pulp regeneration [101]. These studies indicate that scaffold-mediated EV delivery can both protect EV bioactivity and facilitate localised, sustained therapeutic dosing.

Beyond hydrogels, decellularised tissue scaffolds have been explored as EV carriers. Diomede et al. (2022) developed a decellularised dental pulp matrix capable of supporting stem cell repopulation and, when combined with EVs, could potentially provide both structural and biochemical cues for in situ pulp regeneration [101,102]. Such approaches leverage the native ECM to present EVs in a physiologically relevant microenvironment, potentially enhancing cell–matrix and EV–cell interactions.

Innovations in EV functionalisation have also emerged to boost therapeutic efficacy. Surface modification of EVs with targeting ligands, such as RGD peptides, to engage integrin receptors on osteoblasts, has been shown in non-dental models to increase EV homing and uptake by target cells, leading to improved bone regeneration outcomes [103]. Although not yet reported in dental applications, these strategies hold promise for increasing the precision of EV therapies in periodontal and alveolar bone repair.

### 4.2. Overcoming Safety Challenges and Translating EV-Based Therapies to Clinical Practice

To enhance safety and translational potential, EV-based therapeutics must integrate immunogenicity reduction and off-target toxicity mitigation from production through administration. Advanced purification workflows such as tangential flow filtration (TFF) followed by size-exclusion chromatography (SEC) have been shown to produce high-purity EV preparations devoid of xenogeneic proteins and endotoxin contaminants, essential for clinical-grade GMP compliance [104]. Commercial hollow-fibre bioreactor systems utilising xeno-free media further support reproducible EV isolation, yielding high-purity preparations with acceptable safety margins in controlled conditions [104].

Evidence from early clinical case reports suggests that well-matched autologous or screened allogeneic MSC-EVs elicit minimal immunogenicity in human use, as inferred from analogous MSC therapy trials in graft-versus-host disease, where no infusion-related adverse events or dose-limiting toxicities occurred even with repeated dosing [105]. While these outcomes derive from MSC therapy rather than isolated EVs, they provide a supportive rationale to anticipate comparable tolerability in EV applications.

To translate EV therapies into clinical practice, dosing strategies must be grounded in preclinical pharmacokinetics and biodistribution data that define therapeutic windows and off-target accumulation. Dose escalation studies comparing intravenous administration versus localised delivery via scaffolds or hydrogels are necessary to determine the optimal balance between efficacy and safety [106]. These data should be paired with functional potency assays, robust particle and cargo profiling, and regulatory-aligned potency criteria as per MISEV2023 to ensure reproducibility across batches and sites [107].

Enabling safe clinical deployment of EV-based therapies hinges on GMP-compatible purification methods (e.g., TFF-SEC) [108,109], immunologically inert donor matching, and optimised delivery routes informed by rigorous preclinical safety data [110]. Together, these convergence strategies will reduce immunogenic risk, improve off-target control, and support future clinical translation.

### 4.3. Translating EV-Based Therapies to Clinical Practice

Efficient translation of EV-based interventions into dental clinics hinges on establishing robust, GMP-compliant production pipelines. Pachler et al. (2017) demonstrated that BM-MSC expansion in fibrinogen-depleted, pooled human platelet lysate media, with downstream tangential flow filtration and preparative SEC, yields EV preparations exhibiting narrow nanoparticle size distributions, consistent expression of CD9, CD81, and TSG101, and sterility profiles satisfying clinical release criteria [35].

While this scalable approach addresses batch-to-batch variability, further work is needed to define potency assays predictive of oral tissue regeneration, since most current release metrics (particle count, marker presence) do not correlate directly with functional outcomes in alveolar bone or pulp repair [36]. Regulatory classification of EVs is also uncertain: some jurisdictions treat them as biological products, others as cell therapies or drug-delivery systems, each demanding distinct investigational new drug (IND) dossier contents, stability protocols, and post-marketing surveillance plans [37].

Early-phase clinical data in non-dental indications suggest a favourable safety profile. Sengupta et al. (2020) reported that a single 15 mL infusion of ExoFlo, an allogeneic BM-MSC-EV formulation, was well tolerated in severe COVID-19 patients, with no infusion-related adverse events at 72 h, and complementary studies in stage III/IV chronic kidney disease likewise observed no acute toxicity over 12 months [111]. However, these findings cannot be extrapolated to periodontal or endodontic applications, where local scaffold embedding or repeat dosing may produce different biodistribution and immune responses.

Critically, no Phase I/II trials have yet targeted periodontal regeneration or pulp repair using EV therapies. Such studies must define dosing regimens that balance therapeutic efficacy against potential off-target effects in the oral mucosa, compare systemic versus local delivery, and establish therapeutic windows compatible with clinical workflows.

Dose selection for EV therapeutics remains challenging because studies variably report dose as particle number, total protein, or parent cell equivalents, limiting cross-study comparability and rational scaling from animals to humans. A pragmatic approach for translational development is to report dosing in at least one normalised metric (e.g., particles/kg or µg protein/kg) alongside functional potency readouts, enabling batch comparability and dose–response modelling. The route of administration strongly impacts effective exposure; after intravenous delivery, biodistribution studies show preferential accumulation in reticuloendothelial organs, such as the liver and spleen, indicating the possibility that higher systemic doses are required to achieve adequate exposure at oral targets while increasing off-target risk [112]. In contrast, local administration can increase concentration at the intended site and reduce systemic biodistribution. In a mouse biodistribution study, IV dosing led to liver and spleen accumulation, whereas localised delivery routes increased signal in the target compartment and were interpreted to limit extra-organ exposure [112]. These considerations are particularly relevant in regenerative dentistry, where local injection and scaffold or hydrogel delivery are clinically feasible and may permit lower total doses by improving tissue retention and residence time [6]. As such, future dental EV studies should directly compare local versus systemic dosing using standardised reporting in normalised dose units, pharmacokinetic and biodistribution endpoints, and oral-site safety readouts such as local cytokine profiles, gingival histopathology, and effects on mucosal immunity, in addition to regeneration outcomes. Without dental-specific clinical evidence, adoption in routine practice remains premature.

Finally, long-term safety evaluations are indispensable. In murine models, repeat dosing of HEK293F-derived EVs over 30 days resulted in rapid clearance and organ biodistribution profiles comparable to PEG-liposomes, with haematology, serum biochemistry, cytokine assays, and histopathology revealing no immunogenicity or tissue lesions [113]. Wiklander et al. (2015) further refined biodistribution analysis, using DiR labelling, sucrose-gradient purification, and IVIS imaging, to confirm that systemically administered HEK293T-EVs localise primarily to the liver, spleen, GI tract, and lungs in a dose- and route-dependent manner, and that fluorescence derives from intact vesicles rather than free dye [114]. Additionally, the oncogenic potential of EV therapies remains insufficiently studied: to date, no dedicated long-term tumourigenesis assessments have been reported in preclinical models, highlighting a critical gap in safety evaluation that must be addressed before large-scale clinical trials can proceed [115,116].

### 4.4. Ethical and Social Considerations

Clinical investigations of EV therapies in dental and non-dental contexts provide important evidence on tolerability that complements ethical mandates for rigorous oversight. In periodontitis, a proof-of-concept study of non-surgical injections of human platelet-derived EVs in 21 patients (stages I–III) demonstrated significant reductions in probing depth and pro-inflammatory cytokines at six weeks post-treatment, with only transient gingival erythema and no systemic haematological or biochemical abnormalities reported [117]. Alveolar ridge augmentation using the same periosomes formulation restored bone volume successfully and elicited no local or systemic toxicity [118]. In endodontics, a chitosan–hUCMSC exosome hydrogel applied post-pulpectomy facilitated progressive pulp vitality recovery and periapical healing over 24 weeks, with only mild postoperative discomfort noted [119]. These dental findings align with favourable safety profiles in other EV applications; idiopathic facial paralysis treatments reported no functional deterioration or serious adverse effects [120], and combined autologous exosome plus Nd:YAG laser therapy for facial nerve injury achieved marked functional improvements without safety concerns [121].

Ethical frameworks governing EV-based interventions mandate comprehensive informed consent processes, particularly in vulnerable populations, and stringent donor screening and Research Ethics Board approvals to safeguard autonomy and minimise harm [122]. Invasive delivery routes, such as intrathecal or surgical implantation, necessitate transparent patient education on both acute and delayed toxicities [123,124]. Autologous EVs mitigate immunogenic risks but introduce variability and standardisation challenges, whereas allogeneic preparations require protocols akin to cellular therapies to address consent, traceability, and infectious risk [125]. Furthermore, unregulated commercial offerings of human-derived exosome products with misleading safety claims underscore the need for post-market surveillance and enforcement of accurate risk communication [126,127]. Equitable access considerations, from donor compensation frameworks to intellectual property landscapes, must balance fair benefit sharing against undue inducement and monopolistic barriers that could hinder technology transfer and inflate costs in low-resource settings [128,129]. Collectively, integrating clinical safety outcomes with ethical rigour and robust governance will be essential to responsibly advance EV therapeutics in regenerative dentistry and beyond.

The clinical deployment of EV-based therapies must be accompanied by transparent risk communication and robust informed consent processes that fully convey both the known benefits and remaining uncertainties surrounding EV cargo heterogeneity and off-target effects. Lener et al. (2015) emphasise that patient information sheets should include accessible explanations of EV manufacturing (GMP compliance, batch release criteria) and potential immunogenic or biodistribution risks, ensuring that consent is truly informed [130]. Equitable access presents a parallel challenge: high costs associated with GMP-grade EV production and specialised delivery systems risk concentrating advanced therapies in resource-rich centres. An evaluation of cytotherapy economics noted that without novel reimbursement models or tiered pricing strategies, vulnerable populations may be systematically excluded from EV treatments, exacerbating health disparities [131].

Regulatory harmonisation will therefore be crucial not only for safety but also for social legitimacy. The comprehensive review by Japan’s Pharmaceuticals and Medical Devices Agency (PMDA) on EV product quality and safety highlights the need for internationally aligned guidelines on production, characterisation, and post-marketing pharmacovigilance, addressing critical ethical concerns about source material traceability and long-term monitoring of adverse events [132]. Transparent dissemination of clinical trial data and engagement with patient advocacy groups are essential to address misconceptions and inform public understanding [133]. Clear communication of both the therapeutic potential and current limitations of EV-based therapies will be critical for fostering informed acceptance and responsible integration into clinical practice.

## 5. Preclinical and Clinical Studies on EV-Based Therapy in Dentistry

Preclinical and early clinical investigations have validated the regenerative potential and safety of EV-based interventions in dental applications. In rodent and small animal preclinical studies, EVs derived from stem cells and engineered via preconditioning or scaffold integration have consistently provided valuable insights into the biological properties and regenerative potential by showing accelerated dentinogenesis, alveolar bone repair, and periodontal regeneration, while exhibiting minimal overt toxicity (Table 1). Building on efficacy and safety data, human studies, though limited in cohort size, report favourable tolerability and preliminary evidence of clinical benefit in periodontitis and guided bone regeneration procedures (Table 2). The following sections summarise key findings from preclinical investigations and emerging evidence from clinical studies.

### 5.1. Preclinical Studies

Preclinical investigations of EVs in dental tissue regeneration have demonstrated both robust efficacy and an absence of overt toxicity in rodent models. In a rat calvarial defect model, MSCs preconditioned with TNF-α produced EVs that, when applied via a collagen membrane, significantly improved bone regeneration at 4 and 8 weeks, promoted M2 macrophage polarisation, and increased oncostatin M expression, all without evidence of ectopic tissue formation or systemic adverse events [134]. Likewise, in a mandibular alveolar defect model, human dental follicle cell-derived collagenase-released matrix vesicles (CRMVs) outperformed media-collected MVs by restoring approximately 75% bone volume fraction versus 60% in controls, enhancing trabecular architecture, and achieving near-complete defect closure through PLC/PKC/MAPK signalling, again with no signs of local or systemic toxicity [135]. In a mouse periodontitis model, EVs from *P. gingivalis*-LPS-preconditioned dental follicle cells reduced alveolar bone loss, inhibited osteoclast activity, and shifted macrophages toward an anti-inflammatory M2 phenotype, while downregulating apoptotic markers in periodontal ligament cells; animals exhibited no adverse reactions during treatment [136].

**Table 1:** Findings of preclinical studies.

Author [Year]	Source of EVs	Isolation Method of EVs	Route of Administration	Dose of EVs	Animal Model	Main Findings
Huang et al., 2024 [136]	Human dental follicle cells [DFCs] preconditioned with 250 ng/mL *P. gingivalis* lipopolysaccharide [LPS]	Ultracentrifugation from DFC culture supernatant characterised by TEM, NTA, and Western blot [positive for TSG101 and HSP70]	In vivo systemic injection [exact method not specified]; in vitro treatment of human periodontitis-derived periodontal ligament cells [p-PDLCs]	Dose: Not explicitly quantified; in vitro dose optimised for effect	Eighteen 8-week-old male C57BL/6 mice Periodontitis induced by 5–0 silk ligature around the maxillary second molar + *P. gingivalis* inoculation [1 × 10^7^ CFUs per mouse]	In vitro: EVs reduced apoptosis in periodontal ligament cells from periodontitis-affected teeth (p-PDLCs) Downregulated pro-apoptotic markers [Caspase-3, BAX], upregulated anti-apoptotic BCL-2 Decreased RANKL/OPG ratio and inhibited phosphorylation of JNK and P38 In vivo: Reduced alveolar bone loss and osteoclast activity Promoted M2 polarisation [increased CD206, decreased iNOS] Lowered inflammation and bone resorption markers Suggests these EVs modulate inflammation, apoptosis, and osteoclastogenesis via the JNK/P38 MAPK pathway and macrophage polarisation
Kang et al., 2022 [134]	MSCs preconditioned with 20 ng/mL TNF-α [TNFα EVs]; compared with naïve MSC-EVs	Ultracentrifugation; characterised by Western blot [CD63, TSG101, HSP70] and nanoparticle tracking analysis [NTA]	Local application at defect site via collagen membrane soaked with EVs	Not explicitly quantified [standard EV prep used consistently]	Rat calvarial defect model; samples collected at days 1, 3, 7, 28, and 56	EV size and uptake ability were unaffected by TNF-α preconditioning Both EV types enhanced osteogenic markers in MSCs, but TNFα EVs were slightly less effective in vitro TNFα EVs significantly improved bone regeneration at 4 and 8 weeks Promoted M2 macrophage polarisation and reduced M1 markers in vitro and in vivo Increased OSM expression, correlating with bone formation miRNA sequencing showed upregulation of anti-inflammatory miRNAs and enrichment in immunoregulatory pathways [PI3K-Akt, FoxO]
Yi et al., 2022 [135]	Matrix vesicles [MVs] from human dental follicle cells [DFCs]: - Media MVs [MMVs] - Collagenase-released MVs [CRMVs]	MMVs: 7 d osteogenic induction of DFCs Remove cells/debris: 2000× *g* 30 min → 15,000× *g* 1 h 100 kDa ultrafiltration, 0.8 µm filter Ultracentrifuge 150,000× *g* 1 h CRMVs: 7 d osteogenic DFCs → collagenase [300 U/mL, 3 h] Same spin/filter/ultracentrifuge as MMVs	MV-loaded collagen sponge implanted into defect site	1 × 10^9^ particles per scaffold [~40 µg protein]	Rats: 8-wk-old male Sprague–Dawley with 3 mm × 2 mm × 1 mm mandibular alveolar defects [*n* = 4 per group]	Both MMVs and CRMVs internalise into DFCs, enhance proliferation; CRMVs [1 × 10^9^ particles/mL] better promote migration, mineralisation, and upregulate ALP, OCN, OPN, MMP-2; activate PLC-γ1 → PKC → p-ERK/p-p38 cascade. CRMV-loaded scaffolds restored ~75% bone volume fraction vs. ~60% in MMV or scaffold alone [BV/TV]; increased trabecular number/thickness, reduced separation; histology showed near-complete defect closure, robust cortical and trabecular bone; CRMVs outperformed MMVs and matched DFC-seeded scaffolds. Mechanistically, CRMVs—but not MMVs—activated the PLC/PKC/MAPK pathway in recipient cells, underpinning their superior osteoinduction

These studies collectively underscore that EV cargo modulation, whether via inflammatory preconditioning or enzymatic release, can enhance regenerative outcomes in craniofacial bone repair while maintaining favourable safety profiles. However, the variability in dosing regimens, EV isolation methods, and defect models complicates direct comparison and translational planning. Notably, none of the studies reported comprehensive immunotoxicity or biodistribution analyses; future work should integrate multi-parametric safety assessments (e.g., haematology, histopathology, cytokine panels) and explore long-term effects in larger animal systems. Mechanistic insights into macrophage polarisation and MAPK pathway activation provide rational targets for EV engineering, but without standardised potency assays and rigorous off-target evaluations, clinical translation will remain tentative. Table 1 provides an overview of the study models, routes of EV administration, dosages used, and the corresponding responses observed in each study. Key preclinical models, EV sources, isolation methods, administration routes, and dosing metrics are summarised in Table 1.

### 5.2. Clinical Trials and Human Studies

A limited body of clinical research has explored EV-based therapies in dental applications [137]. Notably, a recent proof-of-concept study evaluated non-surgical injections of human platelet-derived EVs in patients (*n* = 21) with stage I–III periodontitis, reporting significant reductions in probing depth and pro-inflammatory cytokines at 6 weeks post-treatment [117]. Safety monitoring across these studies has consistently shown favourable tolerability. In EV studies, only transient gingival erythema occurred in a minority of subjects, with no systemic haematological or biochemical abnormalities reported [117]. In a separate first-in-human case, periosteomes were also used for alveolar ridge augmentation, resulting in successful bone volume restoration and no local or systemic adverse events, marking a milestone in guided bone regeneration procedures [118]. In endodontic applications, a chitosan–hUCMSC-exosome hydrogel used post-pulpectomy promoted progressive pulp vitality recovery and periapical healing over 24 weeks, with only mild postoperative discomfort noted [119].

These findings align with safety data from non-dental EV trials: a pilot study of EVs in idiopathic facial paralysis (Bell’s palsy) observed no functional deterioration or serious adverse effects across seven treated patients [120], and a case series combining autologous exosomes with Nd:YAG laser therapy for facial nerve injury reported marked functional recovery without safety concerns [121].

Despite these positive outcomes, substantial challenges impede the progression of EV therapies in dentistry: most dental studies involve small, non-controlled cohorts, limiting statistical power and generalizability [138]; heterogeneity in EV isolation methods and dosing metrics (ranging from particle counts to protein concentrations) complicates cross-study comparisons and hinders the development of standardised clinical protocols [139]; the optimal route of administration remains unclear (systemic delivery faces rapid clearance by the mononuclear phagocyte system [140], whereas local injections improve site retention but pose technical challenges in uniform distribution within periodontal or pulp tissues [141]); and long-term data on both sustained efficacy and potential delayed adverse effects are lacking, underscoring the need for multicenter, placebo-controlled trials with extended follow-up to elucidate the risk–benefit profile of EVs in regenerative dentistry. Table 2 details the clinical reactions and outcomes of studies using EVs as interventions. In most of these studies, the dosage of EVs was not specified. Available human studies, administration approaches, and reported clinical outcomes are summarised in Table 2.

**Table 2:** Findings of clinical trials.

Author [Year]	Source of EVs	Isolation Method of EVs	Route of Administration	Dose of EVs	Main Findings
Estrin et al., 2025 [118]	Allogeneic EVs [“Periosomes”] combined with bone allograft and platelet-rich fibrin [PRF]	Not specified	Local surgical application within a guided bone regeneration [GBR] membrane during an augmentation procedure	Not specified	CBCT scans at 1, 2, 3, and 6 months showed progressive and significant bone growth. Core biopsy at 3 months confirmed histological alveolar bone regeneration. Implants were successfully placed, demonstrating surgical and histologic success.
Jafari et al., 2025 [119]	Exosomes from human umbilical cord mesenchymal stem cells [hUCMSCs]	Isolation from conditioned medium; confirmed by SEM and TEM [mean size ~101 nm via NTA]	Local application: mixed with chitosan and placed into the root canal following pulpectomy	Not quantified	Single-case report across Bushehr, Shiraz (Iran), and Aktobe (Kazakhstan) on a 40-year-old male with irreversible pulpitis No signs of infection or symptoms over 24 weeks Radiographic imaging indicated periapical healing and periodontal ligament normalisation Clinical signs suggested successful pulp tissue regeneration and tooth vitality preservation
Puletic et al., 2024 [117]	EVs derived from unspecified donor cells [culture source not detailed]—typical of regenerative EV therapies	Not specific	Local injection into periodontal pockets via syringe	Not quantified	Pilot study in Santo André, Brazil (*n* = 14; periodontitis group *n* = 7 [4 M/3 F], stages I–III; healthy controls *n* = 7 [4 M/3 F]); power > 98% at α = 0.05. Pro-inflammatory cytokines, e.g., IL-5 and IL-6, in gingival crevicular fluid were reduced post-treatment to levels comparable to healthy controls. Treatment was safe, well-tolerated, and showed potential for periodontal regeneration

## 6. Regulatory and Quality Control Considerations for EV-Based Therapies

### 6.1. Regulatory Framework

Extracellular vesicle therapies are increasingly viewed by regulators as novel biologics subject to oversight under existing legal frameworks in the United States and European Union. In the U.S., EV-based products are regulated as biological drugs under the Public Health Service Act and the Federal Food, Drug, and Cosmetic Act, which mandate premarket review, compliance with current Good Manufacturing Practices (cGMP), and post-market pharmacovigilance as stipulated by FDA guidance and public safety notifications [142,143]. As of now, no EV therapeutics have achieved FDA approval, prompting enforcement actions and warning letters targeting clinics marketing unapproved exosome treatments without valid biologics licences [144,145]. Similarly, the European Medicines Agency classifies EVs as Advanced Therapy Medicinal Products (ATMPs) under Regulation (EC) No 1394/2007, which requires equivalent manufacturing and clinical standards [144,146,147].

A notable regulatory challenge is the inherent heterogeneity of EV preparations. The variable lipid, protein, and RNA cargo, along with the biogenesis pathways, complicates the establishment of well-defined identity, potency, and comparability assays, as well as detailed pharmacokinetic and biodistribution data [37,148]. Quality control metrics must therefore go beyond particle count and purity to include functional testing and reproducible isolation methods.

Ultimately, translating EV therapies into clinical use depends on integrating robust manufacturing processes, such as scalable isolation, validated cargo profiling, and mechanism-based potency assays, with rigorous regulatory adherence. This holistic quality control approach ensures batch-to-batch consistency, addresses safety concerns inherent in complex biologics, and supports the case for future FDA or EMA approval of EV-based therapies [149].

### 6.2. Quality Control and Standardisation

Reliable therapeutic application of EVs hinges on robust, reproducible isolation and characterisation protocols. Potency assays are quantitative bioassays designed to measure the biological activity of EV preparations, which are directly linked to their intended mechanism of action and clinical indications, such as immunomodulation, angiogenesis, osteogenic, and odontogenic induction. In contrast to identity and purity measurements that include particle concentration, size distribution, tetraspanin and TSG101 expression, and particle-to-protein ratios, potency assays aim to demonstrate that a given batch produces a reproducible and functional effect in a relevant cellular system and can thus serve as a batch-release and comparability tool. In practice, potency testing for MSC-derived small EVs has been approached using mechanism-aligned functional readouts, such as suppression of activated lymphocyte proliferation in mixed-donor mixed lymphocyte reaction (mdMLR) assays as an immunomodulatory potency readout, and endothelial migration and tube formation assays as a pro-angiogenic potency readout. For example, a qualified mdMLR assay has been developed to assess the immunomodulatory activity of EV preparations such as MSC-EVs by quantifying inhibition of T-cell proliferation across multiple donor combinations, improving robustness and reproducibility for potency testing [150]. Similarly, in vitro angiogenesis assays can provide a potency-linked functional readout when EVs are intended to promote neovascularisation, and tube formation responses (network length or branch points) are commonly used to quantify pro-angiogenic activity and support manufacturing comparability [151]. Potency assays can also be tailored to dental indications by selecting readouts that map to therapeutic goals such as macrophage polarisation with M1 to M2 marker shifts, odontogenic and osteogenic differentiation measured by ALP activity and mineralisation, or suppression of inflammatory cytokine output in periodontal ligament-relevant cell systems. Current guidance on MSC-sEV therapeutics also emphasises that potency assays should be fit-for-purpose, standardised for batch release, and ideally anchored to a mechanism that is plausibly causal for the intended regenerative endpoint [152].

Comparative studies have demonstrated marked variability in EV yield and purity depending on the method employed.

Baranyai et al. (2015) compared a rapid 1 h differential ultracentrifugation (UC) protocol to size-exclusion chromatography (SEC) on Sephacryl S-400 columns, demonstrating that UC pellets CD63- and TSG101-positive exosomes together with high levels of co-pelleted albumin, whereas SEC yields the same exosome markers in early fractions with significantly lower albumin contamination [153]. Zhang et al. (2020) evaluated a cascade of PEG precipitation, SEC, and iohexol density gradients, finding that although PEG precipitation followed by SEC isolates EVs efficiently, notable apoA1 and apoB100 contamination remains, and that only the subsequent density gradient step appreciably decreases, but does not entirely eliminate, these non-vesicular particles [154].

To harmonise reporting and facilitate cross-laboratory comparability, MISEV2023 guidelines recommend standardised metrics such as particle concentration (e.g., via nanoparticle tracking analysis), contaminant ratios (e.g., particle-to-protein), and marker panels (e.g., CD9, CD63, TSG101) to define EV preparations [107]. Adoption of well-characterised reference materials, such as GFP-tagged recombinant vesicles for assay calibration, has been shown to streamline high-throughput yield measurements, facilitate rapid process characterisation, and enhance reproducibility in EV isolation workflows [155]. Single-vesicle analyses (e.g., high-resolution flow cytometry, image-based cytometry) and combined zeta-potential/size distribution profiling can sensitively detect residual lipoproteins or protein aggregates that would otherwise elude functional assays [156,157].

EV translation also depends on validated storage and transport conditions that preserve vesicle integrity and function. EVs are sensitive to temperature shifts, adsorption to tube surfaces, and repeated freeze–thaw cycles, which can promote aggregation, alter apparent size distributions, and reduce functional potency even when particle counts appear similar. While −80 °C storage is widely used, current evidence suggests that ideal conditions vary by EV source, buffer composition, and downstream application, and that standardisation remains incomplete. As potential solutions for point-of-care distribution, lyophilisation or freeze-drying has been investigated [158] to reduce cold-chain dependence; however, drying imposes physical stresses that can reduce recoverable particle numbers, unless protective formulations are used. Freeze-drying EVs with lyoprotectants such as trehalose can mitigate particle loss and preserve biological activity compared with unprotected lyophilisation [159], supporting a formulation-dependent route toward room-temperature-stable EV products. Systemic evaluations also emphasise remaining gaps, including EV type-specific limits on freeze–thaw exposure, optimal cryoprotectant selection and concentration, and long-term functional stability under realistic shipping conditions, including temperature excursions, agitation, and delayed processing [160], which require further studies before temperature or storage standards can be established.

At a clinical scale, EV production must adhere to GMP standards, maintaining both purity and potency. Microfluidic platforms, such as viscoelastic flow separation, enable label-free, continuous-flow enrichment of EVs with >90% purity of exosomes, with >80% recovery, while preserving vesicle integrity [161]. Affinity-based capture using anti-CD61 methacrylate-based monolithic columns delivers highly pure platelet-derived EV preparations, achieving a 100-fold increase in particle-to-protein ratio over flow-through and no detectable lipoprotein contamination by FPLC within a 10 min, reusable-disc workflow, though further cross-validation against SEC or density gradient methods is essential before routine adoption [162].

Scalable and clinical-grade EV purification can be increasingly achieved using closed, filtration-based workflows rather than ultracentrifugation. In particular, TFF enables reproducible concentration and buffer exchange from large volumes of conditioned medium or biofluids with reduced shear and improved scalability compared to ultracentrifugation. In a direct comparison, TFF provided a scalable approach for concentrating EVs from large sample volumes while maintaining vesicle integrity and yield [163]. Polishing steps are typically required to improve purity. By combining TFF followed by SEC, this reduces co-isolated protein contaminants while preserving EV-associated markers and functional activity, and comparative technical analyses show that the use of TFF with SEC can achieve purity [164] and yield profiles suitable for downstream functional assays. For upstream functionality, hollow-fibre bioreactor culture supports high-density MSC expansion and continuous harvesting and enables substantially increased EV production compared with conventional flasks [165], which further facilitates GMP-aligned manufacturing when paired with scalable downstream purification.

### 6.3. Risk Management in Clinical Settings Framework

Risk management in clinical EV therapy demands a structured strategy that spans pre-treatment screening, dose and route optimisation, systematic adverse event grading, and extended follow-up. In a single-arm Phase I trial of intrathecal allogeneic human umbilical cord MSC-derived exosomes for subacute spinal cord injury, nine patients underwent baseline neurological and laboratory assessments, received a single infusion, and were followed at 1, 3, 6, and 12 months. Assessments included ASIA motor/sensory scores, SCIM III, NBD evaluation, and CTCAE v5.0 grading. No early or delayed adverse events were attributed to the intervention over one year, demonstrating strong tolerability in a small cohort [124]. This clinical experience aligns with broader findings that intrathecal cell or exosome administrations typically show acceptable safety profiles in human neurological trials.

Another study in refractory Crohn’s perianal fistula patients applied a multimodal monitoring approach over six months, encompassing initial in-hospital surveillance for acute cardiac, respiratory, or allergic events; six-hourly labs during early post-administration, and weekly outpatient follow-up, with no serious adverse events observed [130]. These structured designs highlight that effective risk mitigation hinges on integrated clinical, laboratory, and functional monitoring aligned with standardised criteria like CTCAE and regular clinical reviews to detect both overt and subclinical toxicities. However, most published EV trials remain limited by small sample sizes and the absence of independent data and safety monitoring boards, which are standard in oncology and other therapeutic areas, to review unblinded safety data and define dose-limiting toxicity.

To strengthen safety frameworks in future EV trials, adopting guidance elements from clinical research best practices, such as risk-based monitoring outlined in the European Medicines Agency guidelines on human medicines [166], trial master file documentation, and data and safety monitoring oversight, is essential [167]. Implementation of a data and safety monitoring plan proportionate to trial complexity, interim safety reviews, and explicit escalation criteria would preserve patient safety and trial integrity. Post-market pharmacovigilance systems like FDA MedWatch or EMA EudraVigilance are also critical to identify rare or late-onset adverse events as EV therapeutics become more widely used [168]. While existing phase I trials demonstrate potential for EV safety, a robust risk management infrastructure, with independent oversight, rigorous adverse event grading, and long-term follow-up, will be vital for safe scaling of EV-based therapies into broader clinical use.

## 7. Conclusions

Although this review provides a comprehensive synthesis of the available evidence on the safety of EV-based therapy in regenerative dentistry, several limitations must be acknowledged. Firstly, the search strategy was focused on preclinical and clinical studies within dental applications, which may have excluded relevant data from broader regenerative fields that could provide valuable mechanistic insights. Secondly, the heterogeneity in study design, EV sources, isolation methods, and outcome assessments limits direct comparison across studies and introduces potential selection and reporting bias. Furthermore, the reliance on small animal models, limited long-term follow-up, and underreporting of adverse events in several studies may obscure true safety profiles. Finally, the current lack of standardisation in EV characterisation, as emphasised by adherence variability to MISEV and GMP guidelines, further challenges translational interpretation. Despite these limitations, existing studies collectively suggest that EVs exert regenerative effects primarily through modulation of inflammation, stimulation of angiogenesis, and promotion of progenitor cell recruitment and differentiation. In periodontal models, MSC- and PLT-derived EVs attenuated inflammatory cytokine expression and enhanced tissue regeneration by upregulating osteogenic markers.

EV therapies hold transformative potential for regenerative dentistry, offering a safe and effective cell-free approach to tissue repair. While preclinical evidence is compelling, the field must overcome challenges in standardisation, safety validation, and clinical scalability. With continued interdisciplinary research and regulatory alignment, EV-based strategies are poised to become a cornerstone of future dental regenerative protocols.

## Data Availability

No new data were created or analysed in this study. Data sharing is not applicable to this article.

## Abbreviations

The following abbreviations are used in this manuscript:

ABBMC	Anorganic Bovine Bone Mineral with 10% Collagen
AKT	Protein Kinase B [AKT]
ALT	Alanine Aminotransferase
ApoB	Apolipoprotein B
ApoE^−^/^−^	Apolipoprotein E-Deficient
ASIA	American Spinal Injury Association
AST	Aspartate Aminotransferase
ATMP[s]	Advanced Therapy Medicinal Product[s]
BCL2	B-cell Lymphoma 2
bFGF	Basic Fibroblast Growth Factor
BMP	Bone Morphogenetic Protein
BMP-2	Bone Morphogenetic Protein 2
BUN	Blood Urea Nitrogen
C1q/C1s	Complement Component 1q/1s
C3	Complement Component 3
C3a	Complement Component 3a
CBC	Complete Blood Count
CBCT	Cone Beam Computed Tomography
CTCAE v5.0	Common Terminology Criteria for Adverse Events Version 5.0
DAMP[s]	Damage-Associated Molecular Pattern[s]
DEX	Dendritic Cell-Derived Exosomes
DFSC MVs	Dental Follicle Stem Cell-Derived Matrix Vesicles
DFSC	Dental Follicle Stem Cell
DMP1	Dentin Matrix Protein 1
DOK	Dysplastic Oral Keratinocyte Cell Line
DPSC[s]	Dental Pulp Stem Cell[s]
DSPP	Dentin Sialophosphoprotein
EC	European Commission
EMA	European Medicines Agency
ERK	Extracellular Signal-Regulated Kinase
EV[s]	Extracellular Vesicle[s]
Exo	Exosome
FDA	U.S. Food and Drug Administration
FPLC	Fast Protein Liquid Chromatography
FXa	Activated Factor X
GATA2	GATA Binding Protein 2
GBR	Guided Bone Regeneration
GFP	Green Fluorescent Protein
GMP	Good Manufacturing Practice
GMSC	Gingival Mesenchymal Stem Cell
HEK293T	Human Embryonic Kidney 293T Cell Line
HGC-27	Human Gastric Cancer Cell Line 27
hiPSC	Human Induced Pluripotent Stem Cell
HMGB1	High Mobility Group Box 1
H-PRF	Horizontal Platelet-Rich Fibrin
HUC MSC/ hUCMSC	Human Umbilical Cord Mesenchymal Stem Cell
HUVEC	Human Umbilical Vein Endothelial Cell
ICAM-1	Intercellular Adhesion Molecule 1
IgG	Immunoglobulin G
IL-1β	Interleukin-1 Beta
IL-6	Interleukin 6
IP-10	Interferon Gamma-Induced Protein 10
iPSC	Induced Pluripotent Stem Cell
IκBα	Inhibitor of Nuclear Factor Kappa B Alpha
KLF4	Kruppel-Like Factor 4
LAL	Limulus Amebocyte Lysate
Lin28B	Lin-28 Homologue B
lncRNAs	Long non-coding RNAs
LPS	Lipopolysaccharide
MAGE	Melanoma-Associated Antigen
MAPK	Mitogen-Activated Protein Kinase
MDC	Macrophage-Derived Chemokine
MG63	Human Osteosarcoma Cell Line
MIP-1α	Macrophage Inflammatory Protein-1 Alpha
miR	microRNA
MISEV	Minimal Information for Studies of Extracellular Vesicles
MSC[s]	Mesenchymal Stem Cell[s]
NBD	Neurogenic Bowel Dysfunction
Nd:YAG	Neodymium-Doped Yttrium Aluminium Garnet [Laser]
NF-κB	Nuclear Factor Kappa B
NTA	Nanoparticle Tracking Analysis
OCT4	Octamer-Binding Transcription Factor 4
PCNA	Proliferating cell nuclear antigen
P.g. LPS	*Porphyromonas gingivalis*-Derived Lipopolysaccharide
P.g.	*Porphyromonas gingivalis*
PDGF	Platelet-Derived Growth Factor
PDGF-BB	Platelet-Derived Growth Factor BB
p-PDLCs	Periodontal ligament cells from periodontitis-affected teeth
PDLSC-Exo	Periodontal Ligament Stem Cell-Derived Exosomes
PDLSC	Periodontal Ligament Stem Cell
PEG	Polyethylene Glycol
PI3K	Phosphoinositide 3-Kinase
PKC	Protein Kinase C
PLT	Platelet
PPL	Procoagulant Phospholipid
PS	Phosphatidylserine
RAGE	Receptor for Advanced Glycation End-Products
Rictor	Rapamycin-Insensitive Companion of mTOR
RUNX2	Runt-Related Transcription Factor 2
SATB2	Special AT-Rich Sequence-Binding Protein 2
SCAP-Exo	Stem Cells from the Apical Papilla-Derived Exosomes
SCAP	Stem Cells from the Apical Papilla
SCC15	Squamous Cell Carcinoma Cell Line 15
SCIM III	Spinal Cord Independence Measure, Version III
SEC	Size-Exclusion Chromatography
SEM	Scanning Electron Microscopy
sEV[s]	Small Extracellular Vesicle[s]
SGC7901	Gastric Cancer Cell Line
SHED-Exo	SHED-Derived Exosomes
SHED	Stem Cells from Human Exfoliated Deciduous Teeth
siRNA	Small Interfering RNA
Smad	Suppressor Of Mother Against Decapentaplegic Family Proteins [TGF-β Signal Transducers]
SOX2	SRY-Box Transcription Factor 2
TEM	Transmission Electron Microscopy
THBS2	Thrombospondin-2
TLR4	Toll-Like Receptor 4
TNF-α	Tumour Necrosis Factor Alpha
TSG101	Tumour Susceptibility Gene 101
VCAM-1	Vascular Cell Adhesion Molecule 1
VEGF	Vascular Endothelial Growth Factor
VEGFR	Vascular Endothelial Growth Factor Receptor
VEGFR2	Vascular Endothelial Growth Factor Receptor 2
vWF	von Willebrand Factor
WBC	White Blood Cell Count
Wnt	Wingless/Integrated Signalling Pathway
β-catenin	Beta-Catenin
